# Digital Social Media, Youth, and Nonmedical Use of Prescription Drugs: The Need for Reform

**DOI:** 10.2196/jmir.2464

**Published:** 2013-07-26

**Authors:** Tim K Mackey, Bryan A Liang, Steffanie A Strathdee

**Affiliations:** ^1^Institute of Health Law StudiesCalifornia Western School of LawSan Diego, CAUnited States; ^2^School of MedicineDepartment of AnesthesiologyUniversity of California, San DiegoLa Jolla, CAUnited States; ^3^San Diego Center for Patient Safety - School of MedicineDepartment of AnesthesiologyUniversity of California, San DiegoSan Diego, CAUnited States; ^4^Division of Global Public HealthDepartment of MedicineUniversity of California, San DiegoSan Diego, CAUnited States

**Keywords:** non-medical use of prescription medications (NUPM), eHealth, Internet, social media, youth and adolescents, drug abuse, substance abuse

## Abstract

The tragic death of 18-year-old Ryan Haight highlighted the ethical, public health, and youth patient safety concerns posed by illicit online nonmedical use of prescription drugs (NUPM) sourcing, leading to a federal law in an effort to address this concern. Yet despite the tragedy and resulting law, the NUPM epidemic in the United States has continued to escalate and represents a dangerous and growing trend among youth and adolescents. A critical point of access associated with youth NUPM is the Internet. Internet use among this vulnerable patient group is ubiquitous and includes new, emerging, and rapidly developing technologies—particularly social media networking (eg, Facebook and Twitter). These unregulated technologies may pose a potential risk for enabling youth NUPM behavior. In order to address limitations of current regulations and promote online safety, we advocate for legislative reform to specifically address NUPM promotion via social media and other new online platforms. Using more comprehensive and modernized federal legislation that anticipates future online developments is critical in substantively addressing youth NUPM behavior occurring through the Internet.

## Introduction

On February 12, 2001, Ryan Haight, an 18-year-old honors student and varsity athlete from California, USA, died from an overdose of the opioid prescription drug Vicodin (hydrocodone/acetaminophen) bought from an online pharmacy without a prescription [1]. His death highlighted the immediate patient safety and public health risks of nonmedical use of prescription medicines (NUPM) by youth (ie, children and adolescents) obtained from the illicit online environment. This tragic event led to passage of the 2008 US federal legislation, the Ryan Haight Online Pharmacy Consumer Protection Act (RHA), which established regulatory provisions and tools for the Drug Enforcement Agency (DEA) of the United States to control the sale and dispensing of controlled substances over the Internet [2].

However, the effectiveness of the RHA on NUPM online sourcing and regulation of online pharmacies has not been well established or studied. Consequently, the problem of illicit online sourcing of controlled substances and other medications without a prescription has yet to be adequately resolved [1,3]. Despite RHA passage, new forms of unregulated digital media and information technology platforms continue to be developed and are rapidly becoming associated with illicit online prescription drug sourcing in digital environments highly populated by youth.

In order to inform policy efforts to address youth NUPM and current regulatory limitations, we explore the potential public health and patient safety implications of promotion of youth-based NUPM in social media. To do so, we first review current national trends in youth NUPM behavior and Internet and social media utilization. We then examine the use of social media by illicit online pharmacies in promoting NUPM and analyze current policy instruments, including the RHA. We then recommend policy solutions and advocate for additional research to better inform the public and ensure safe Internet access to prevent youth NUPM.

## Nonmedical Use of Prescription Drugs

### National Trends in NUPM

Since Ryan Haight’s death, prescription drug abuse among youth has become part of a larger national trend of morbidity and mortality associated with drug overdose, diversion, and polydrug abuse [4-7]. The US Centers for Disease Control and Prevention (CDC) reported in 2010 that more than 12 million people engaged in nonmedical use of prescription painkillers alone; misuse/abuse of this drug class was responsible for approximately 475,000 emergency room admissions in 2009 [6,8]. Indeed, misuse has led to a marked increase in US public and private health care expenditures, estimated up to $72.5 billion in direct costs annually [8-10]. Prescription drug abuse also disproportionately impacts vulnerable populations, including rural groups, low-income groups, those subject to sexual victimization or dating violence, those with a history of mental illness, and those with a history of substance abuse disorders [5,6,11-13].

### Youth NUPM

Crucially, a key high-risk group for NUPM is youth (children and adolescents, aged 12-17). Estimated prevalence of NUPM among this age group is high, with the CDC reporting in 2011 that 20.7% of high school students had engaged in NUPM (OxyContin, Percocet, Vicodin, Adderall, Ritalin or Xanax) [14]. A 2010 National Survey on Drug Use and Health similarly reported at least 3.0% of all youths (and 5.9% of 18-25 year olds) reported psychotherapeutic NUPM in the past month in 2010 [4,15]. Other studies report even higher prevalence of abuse [12,16]. More recently, the Monitoring the Future 2011 national survey reported that after marijuana, prescription and over-the-counter medications represented the most commonly abused drugs for either licit or illicit drugs among 12^th^ graders [17]. Most troubling, NUPM use among youth may also lead to other forms of substance and illicit drug abuse [4,7,18-20].

Youth narcotic and controlled substance NUPM, which includes commonly abused pain medications such as OxyContin and Vicodin, is perhaps the most deeply concerning risk to youth health development and has even been associated with illicit heroin drug abuse [1,21]. NUPM in these drug classes can result in severe adverse clinical interactions and side effects, drug dependency, and increased emergency room admissions [2,10,16,22]. NUPM is also associated with other high-risk health behavior including alcohol consumption and marijuana use, resulting in poor school performance—yet may be perceived as a lower risk behavior by youth compared to other forms of illicit drug abuse [1,3,12,15,17]. Additionally, increases in NUPM associated with Attention Deficit Hyperactivity Disorder drugs (ADHD), such as Ritalin and Adderall, have become a serious concern [22-25]. Importantly, virtually all these drugs have been detected as marketed by illicit “no prescription” online pharmacies and have been subject to counterfeiting [1,2,25-28].

### Traditional NUPM Sourcing

Traditional methods of drug diversion, including person-to-person purchasing, trading, loaning, sharing, stealing and theft, family member and friend access, street drug purchases, prescription forgeries and fraud, and “doctor/prescription shopping”, have traditionally enabled NUPM [1,12,16]. In order to address these vulnerabilities, some US states have implemented “Prescription Drug Monitoring Programs” (PDMPs) to track prescribing and dispensing of controlled substances in order to detect suspected abuse and diversion [4-7,16].

Although these programs may provide controls to stem diversion of high-risk prescription drugs to youth populations [6,8,16], they are highly uneven in enforcement and state resource commitment [29]. Consequently, they may be ineffective for broader identification and intercession in youth NUPM sourcing. But further, uneven PDMPs may not be responsive to the changing nature of health information seeking and online behavior associated with youth NUPM. Indeed, PDMPs may miss the mark in terms of where youth NUPM sourcing is starting to occur and do not address online sourcing of prescription drugs, which may be familiar to youth yet difficult to trace for illicit activity [22,30-32]. To date, this specific risk factor has not been adequately assessed in youth-related NUPM research.

## Potential Online Risks for NUPM Behavior

### Internet and Social Media Utilization Trends

Exacerbating risks of NUPM access is unregulated content on the Internet, the use of which is now ubiquitous among both youth and adults. Indeed, survey data from the Pew Research Center’s Internet and American Life Project (Pew Internet) indicate that some 72% of US adult Internet users search for health and medical information online and that more than one third engage in health care self-diagnosing [33,34]. In addition, the US Food and Drug Administration (FDA) reports 23% of adult Internet consumers have admitted to purchasing a prescription medicine online, of whom 15% acknowledged the risky nature of purchasing from an online pharmacy located outside the United States [35].

As might be expected, Internet use by the youth demographic is almost universal. Pew Internet reports that an estimated 95% of teens (ages 12-17) [36] currently use the Internet and are the most likely age groups to have an online presence [37]. In addition, there has been a rapid rise in utilization of social networking reflected by a majority (80%) of online teens using popular social media sites including Facebook (93%), and use of other social media platforms including Myspace (24%), Twitter (12%), and YouTube (6%) [36]. Indeed, youth respondents have reported that the Internet is their primary source of general information, even if the credibility of such information is difficult to determine [38].

Although this population group has widespread adoption of the Internet and social media, they may not engage in safe online behavior. For example, at least 44% of teens admitted they lie about their age to access websites or to set up an online account [36]. Indeed, those using social media sites report being twice as likely as nonusers to misrepresent their age [36]. At the same time, teens are reporting that they use online sources for looking up health, dieting, and physical fitness information (31%) and that 17% of them go online for information on difficult topics including drug use and sexual health [37].

Within this already vulnerable population, there is also a disproportionate income effect. Teens from lowest-income families are twice as likely (23% vs 11%) to seek health information online compared to teens from higher income households [37]. Further, almost half (48%) of teens report purchasing items online, indicating that teens may be comfortable and have access to make potentially illicit purchases if appropriate controls are absent [37].

### NUPM and Illicit Online Pharmacies

Youth online behavior trends indicate that this population is adopting digital technology for consumption of health information and may be engaged in risky online behavior, which can increase risk for Internet-enabled NUPM [37,39]. Several studies have identified the public health risks of sourcing from “no prescription” illicit online pharmacies that enable NUPM, including among youth and adolescents [9,22,26,27,30,32,40,41].

Importantly, any online pharmacy purportedly marketing the sale of a prescription medication without the need of a prescription is both violating applicable US laws and regulations, as well as promoting NUPM behavior given that adequate controls to ensure patient safety are lacking. This promotion of NUPM is often facilitated by false and misleading marketing used in online direct-to-consumer advertising (DTCA) [42,43], which has yet to be adequately regulated by FDA and others [42,43]. These illicit forms of DTCA may be difficult for consumers, particularly youth, to accurately identify as legitimate (or not), despite public service announcements attempting to inform consumers that online purchasing can be dangerous [44].

Despite its illegality, the spectrum of drugs available for online NUPM sourcing is virtually unlimited [1]. This includes a host of therapeutic drug classes marketed without sufficient controls, including drugs for weight loss, ADHD, steroids, inhalants, contraception drugs and devices, opioids, a variety of narcotics, and drugs in critical shortage promoted across various Internet mediums, including social media [9,22,24,26,30,32,40,41,43,45,46].

Collectively, these studies illustrate that illicit online sourcing represents a potential risk factor for youth NUPM. Illicit NUPM promotion through Internet pharmacies engenders a completely unregulated system of parallel access for youth. This can lead to self-prescribing of virtually any medicine, resulting in drug abuse and dependence, as well as use of drug forms that are of questionable quality, authenticity, and safety, all without medical or parental oversight [1,28]. Tragically, this form of NUPM sourcing has been directly linked to patient deaths, including Ryan Haight, as well as others [1,28].

### Lack of Sufficient Research on Social Media and NUPM

Recognition and needed research on the convergence of social media and youth NUPM is highly uneven. Despite growing evidence of online sourcing risks, a recent systematic review of NUPM behavior among adolescents failed to mention online information seeking/sourcing or social media usage as a specific risk factor [12]. Conversely, organizations such as the National Center on Addiction and Substance Abuse have *specifically* identified increased risks associated with substance abuse for youth who use social media [39]. The United Nations International Narcotics Control Board also warns that illicit Internet pharmacies have started using social media to target young audiences [47].

Some studies have also attempted to assess this area of risk. Previous research has identified increasing use of popular social media platforms by illicit “no prescription” online pharmacies marketing the sale of several high-risk drug products [24,43,46,48]. This includes a recent study that found that illegal DTCA marketing of a fictitious illicit online pharmacy using social media sites Facebook, Myspace, and Twitter was easily accessible and could be done at low cost [48].

Another published study examined the use of Twitter to discuss Adderall NUPM behavior among college students [49]. It found that 8.9% of Adderall-related tweets analyzed mentioned another substance (including illicit drugs), indicating the dangerous possibility of promotion of polydrug abuse via social media [49]. Another unpublished study analyzed Adderall-related Twitter traffic and found that the highest volume of Twitter content (roughly 7 out of 10) originated from illicit online pharmacies advertising the sale of medications with no prescription required [50].

Though an evidence base supporting the association between social media and NUPM is beginning to emerge, there is an urgent need for additional research specifically examining in detail NUPM-related risk factors enabled by social media. This should be pursued in conjunction with policy analysis to determine if current law and legislation can effectively regulate this digital medium to ensure youth and patient safety.

### Ineffective Enforcement/Coverage of Existing Regulations

More than 10 years after Ryan Haight’s unintentional death, youth online-enabled NUPM access remains relatively unabated despite legislative and law enforcement efforts. Global action (such as Interpol’s Operation Pangea) have led to the closure of some illicit online pharmacies [51]. Yet despite these operations, organizations such as the National Association of Boards of Pharmacy (NABP) continue to report that the vast majority (97%) of existing online pharmacies are “not recommended” and present potential patient safety risks [52]. This includes 87% of recent NABP-reviewed online pharmacies not requiring a valid prescription for dispensing [52].

The specific mechanisms of the RHA to stem controlled substance online NUPM focus on registration, licensure, disclosure, and reporting requirements for online pharmacies offering controlled substances as well as requiring valid prescriptions for dispensing (including at least one in-person examination) [2]. It also imposes increased penalties for illicit actors in an attempt to deter such criminal activity [2]. Yet, the RHA primarily focuses on domestic online pharmacies, which is problematic given that surveys have identified up to 23% having a physical addresses outside the United States and most do not provide any address at all [1,52]. Hence, online pharmacies selling controlled substances that operate outside of the United States may not be subject to the jurisdiction of the Act or the DEA, limiting enforceability.

Additional gaps in the RHA in effectively dealing with the illicit online sale of controlled substances have also been reported. This includes websites “unlocking” hidden content that provides access to controlled substances and using affiliate networks and portal sites to avoid law enforcement detection [3]. Further, other illicit actors may simply sell the “prescription” to the patient for an additional fee, allowing for re-use and may not be subject to the RHA (1). Criminals operating online pharmacies have also gone as far as impersonating DEA agents and defrauding consumers by threatening law enforcement and prosecution for illegal purchase of a drug after a consumer has purchased online [53].

Further highlighting the limitations of the RHA in effectively regulating controlled substance NUPM, a 2011 report by online monitoring company LegitScript, published a sample list of 1000 illicit online pharmacies actively offering the sale of controlled substances without a valid prescription (including over half with domain name or server presence in the United States)—an activity in direct violation of the RHA [3]. Yet, despite these clear legal violations and claims by DEA of RHA effective deterrence, there appears to be little enforcement with no successful prosecutions under the RHA against these or other criminal violations of the law [3,54]. Hence, there is a clear need to reexamine the scope and coverage of the RHA and enable additional tools of enforcement to meet changing online trends and current regulatory gaps.

## Reform

### Amending the Ryan Haight Act

Associated risks of NUPM to patient safety and public health are high, but in no group is the risk greater than in youth and adolescents. The physical, mental health, and emotional harms from youth NUPM can have lasting impacts for this vulnerable population [17]. Yet, the combination of the continuing national public health crisis of youth NUPM, increased utilization by youth of the Internet and social media, and an insufficiently regulated online environment that allows NUPM promotion and sourcing continue to put youth at significant risk. Though illicit online pharmacies that enable NUPM behavior present a global public health problem requiring international cooperation, even at the domestic level, amendments to the existing RHA could improve effectiveness and enforceability to better prevent youth NUPM.

Reform should begin with examining amendment and modernization of the RHA to improve its scope, effectiveness, and enforceability over illicit online NUPM promotion of prescription controlled substance drugs where it is actively occurring. First, the RHA does not specifically address other nonInternet pharmacy actors that actively facilitate this illicit trade. These Internet service intermediaries are clearly enabling NUPM behavior and sourcing and may also profit from this illicit activity through generation of revenue from search engine marketing/optimization, ad revenue, and processing, membership, and referral fees [1,43]. Specifically, the RHA does not address NUPM promotion through social media, though these forums have already been identified as allowing promotion of NUPM by illicit online pharmacies [43,48,49].

These enabling risk factors require RHA amendment to expand its scope and enforcement powers to address new forms of digital communication and media that promote online NUPM. This could be accomplished by amending the RHA to include a new definition of “Enabling 3^rd^ Party Intermediaries” to capture additional and relevant online mediums promoting NUPM and illicit access points. Through amending the RHA to include this provision, this term can encompass online digital technologies, including nonpharmacy websites, Internet service providers (ISPs), Web applications, mobile-based platforms/games, payment processors, affiliate sites, membership forums, and, specifically, social media sites. Further, it can focus on high-risk and clearly illegal online promotion activities that advertise sourcing without a prescription, facilitate NUPM sourcing (through direct links to online pharmacies, online ads, etc), or fail to monitor and remove direct marketing associated with NUPM promotion often in direct violation with their own legal terms of conditions and use (including key social media platforms) [48].

Indeed, despite potential facilitation of illicit sourcing, third-party sites have remained largely unregulated and have for the most part escaped enforcement efforts [28]. One clear exception has been the world's largest search engine Google, which was fined $500 million by the US Department of Justice in 2011 for illegal online pharmacy ads that led to a change in its AdWords program [55]. Hence, by pursuing amendment of the RHA, if any of these third-party intermediaries in fact have a physical location or infrastructure in the United States, jurisdiction could be extended over them and their actions could be made subject to the enforcement provisions of the Act [43]. In this way, the entire digital ecosystem of online-enabled NUPM can be addressed through simple amendment of existing legislation enabling the DEA to pursue more proactive enforcement actions to promote public health.

In addition, in order to provide consumers with important and necessary information on safe online sourcing of controlled substances, the RHA should also be amended to require the DEA to publish a publicly available list of online pharmacies that have successfully modified their DEA registration to allow online sale of controlled substances as statutorily required under the Act [2]. This list of authorized and registered DEA online pharmacies should also incorporate with NABP verification through its Verified Internet Pharmacy Practice Sites (VIPPS) program, the only system recommended by the FDA. This would better ensure ongoing RHA compliance and state licensure verification and better inform consumers about safe online sourcing. Additionally, use of monitoring companies such as LegitScript, which has clients such as Google, Microsoft, Amazon, and the FDA, may better ensure that authorized sites are actively monitored and remain compliant with RHA mandates.

### Lessons From Past Legislation

Past failed legislative efforts to more dynamically regulate online pharmacies may provide important lessons for future potential solutions. In 2012, two US congressional bills, the House’s Stop Online Piracy Act and the Senate’s Protect IP Act, included provisions to regulate domestic and foreign online pharmacy websites, and associated search engines, payment processors, and other ISPs, that facilitate illicit online drug e-commerce [56]. However, these bills also contained additional intellectual property rights provisions for other forms of digital medium and online services (eg, videos, music, etc) not related to health that became the subject of controversy and protest and led to the defeat of both bills [56].

In retrospect, it seems clear that important public health considerations to protect consumers online need to be positioned in their own unique legislation that solely addresses issues of patient safety and does not concurrently address commercial or intellectual property rights. Hence, an amendment of the RHA may provide for such a policy forum, as controlled substance NUPM among youth and adolescents continues to represent a national public health crisis that existing law has arguably failed to adequately address and the subject is sufficiently narrow in scope compared to general anticounterfeiting legislation. Though legislative action may face challenges, amendment of the RHA could modernize the Act to respond to emerging digital technologies, provide additional tools to the DEA in pursuing enforcement, and address regulatory gaps currently being exploited by illicit online pharmacies

### Increased Policy Advocacy and Action

Last, there is a need for better cooperation and tangible action by stakeholders currently advocating for action against illicit online pharmacies. The Center for Safe Internet Pharmacies (CSIP), a nonprofit organization with goals of combating illegal online pharmacies through education, enforcement, and information dissemination, was formed in 2011 and is a partnership between numerous private sector entities actively involved in e-commerce [57].

Included in CSIP membership as a strategic partner and board member is the world’s largest social media platform, Facebook [58]. Yet, despite its membership and apparent public engagement on this issue, recent research indicates that social media sites do little to enforce their own policies or monitor their content for NUPM-related promotion [48]. CSIP and other organizations, such as the Association of Safe Online Pharmacies, must more actively engage member ISPs and other stakeholders they partner with to prioritize accountability and enforcement against clearly illicit NUPM promotion, especially that which targets youth.

## Conclusions

The frenetic pace of technology change through new forms of digital sources has quickly made existing legislative approaches to maintain online drug safety antiquated. This is reflected in today’s “Ryan Haight”, who is not only frequently on the Internet but is also a common if not daily user of popular social media sites such as Facebook, a platform already linked to NUPM promotion [24,43,48]. He or she may also be in any part of the world with Internet access and subsequently has access to a global illicit online trade of suspect medicines that bypasses country borders and rule of law.

Hence, it is crucial that particularly youth, who are already at high risk of NUPM and are the most active demographic on the Internet, be provided a safe online environment to make rational and informed choices not to engage in dangerous health behavior. Unfortunately, the present environment presents significant challenges for this important decision-making process and attempts at prevention. Hence, domestic and international approaches addressing NUPM must be modernized to meet the needs of a new digital youth generation and prevent the unnecessary death of the next Ryan Haight.

## Abbreviations

ADHD: Attention Deficit Hyperactivity Disorder
CDC: US Centers for Disease Control and Prevention
CSIP: Center for Safe Internet Pharmacies
DEA: US Drug Enforcement Agency
DTCA: direct-to-consumer advertising
FDA: US Food and Drug Administration
ISPs: Internet Service Providers
NABP: National Association of Boards of Pharmacy
NUPM: nonmedical use of prescription medicines
PDMPs: Prescription Drug Monitoring Programs
Pew Internet: Pew Research Center’s Internet and American Life Project
RHA: Ryan Haight Online Pharmacy Consumer Protection Act
VIPPS: Verified Internet Pharmacy Practice Sites
